# The Effect of Sociodemographic and Anthropometric Variables on Nutritional Knowledge and Nutrition Literacy

**DOI:** 10.3390/foods13020346

**Published:** 2024-01-22

**Authors:** Nevin Sanlier, Funda Kocaay, Sule Kocabas, Pinar Ayyildiz

**Affiliations:** Department of Nutrition and Dietetics Ankara, Faculty of Health Sciences, Ankara Medipol University, Ankara 06050, Turkey

**Keywords:** health, nutrition education, nutrition knowledge, nutrition literacy, sociodemographic variables, anthropometric measures

## Abstract

Nutrition literacy, which is one of the important components of health literacy, includes basic nutritional information and understanding, interpreting and having the ability to make healthy decisions on nutrition-related issues. This study aims to dwell upon the relationship between sociodemographic and anthropometric variables and nutritional knowledge and nutrition literacy. A total of 1600 people aged 19–64 years, 934 women and 666 men, voluntarily participated in the research in the capital city of Turkey. The mean age is 28.2 ± 10.9 years. More than half of the participants (57.4%) have a university graduate/postgraduate education level, and 66.2% are unemployed. This cross-sectional study evaluated demographic information, anthropometric measurements, nutritional information and nutrition literacy. Nearly all the respondents (94.6%) were determined to have sufficient nutrition literacy. Body mass index (BMI) and age were negatively associated with nutrition literacy, whilst nutrition knowledge was positively associated. Respondents with nutrition education at school had the highest nutrition knowledge and nutrition literacy scores, and primary school graduates had the lowest. Participants who received nutrition education scored higher in all the subgroups of the GNKQ. Age, gender, marital status, education status, employment status, BMI and nutrition education were significantly associated with nutrition literacy. The results will be useful in developing food and nutrition policies that will pave the way for making decisions on the most useful themes of health and nutrition campaigns.

## 1. Introduction

Nutritional behavior is a highly complex phenomenon that is influenced by various factors such as nutrition knowledge [1]. It is known that effective and continuous nutrition education plays an important role in the protection and development of health; it is fundamental for changing wrong dietary habits of all ages [2]. When it comes to health-enhancing behaviors, health literacy is considered one of the most important predictive factors [3]. Insufficient health literacy limits the recognition of health problems [4]. Nutrition literacy, which is one of the important components of health literacy, is the ability to understand, interpret and access the services and basic nutritional information to decide on nutritional issues [5]. It is known that knowledge, attitudes, skills and behaviors about food and nutrition affect food selection [6,7,8]. Increasing evidence suggests that most people have difficulty using the information on food labels, and those with insufficient health literacy and/or mathematical skills face worse health outcomes [9,10]. Moreover, nutrition literacy has three different components: functional nutrition literacy (FNL), interactive nutrition literacy (INL) and critical nutrition literacy (CNL). FNL is defined as an individual’s capacity to understand and grasp nutritional concepts and nutritional messages, INL is defined as the cognitive skills required to cope with nutritional problems, and CNL is defined as the capacity of an individual to evaluate nutritional information and to critically address nutritional problems and barriers between peers and the social environment [1,11]. Nutrition literacy is vital for individuals, especially in regions where there is inequality in nutrition, health and education [12]. The stages of reaching the right information, interpreting and making the right decision are critical for sustainable healthy eating behaviors [13]. Therefore, to reduce the increasing prevalence of NCDs and to improve life quality, healthy eating knowledge and behaviors should be developed [14,15].

Holding a highly complex entity influenced by socio-cultural, financial, medical and genetic-related factors, nutritional behavior in particular is worth scrutinizing from a societal perspective. To this end, the number of studies intending to shed light on the components of nutrition literacy such as socio-demographic elements seem to have elevated in international literature, yet this is not the case for Turkey at present, which points to a call for research in this regard. Designing these studies in question would be a highly strategic act vis-à-vis protecting public health and eschewing diseases through enhancing nutrition literacy, especially taking into account the links between nutrition literacy, nutrition knowledge and public health. The aim of this study is to determine the relationship between sociodemographic variables and nutritional knowledge and nutrition literacy.

## 2. Materials and Methods

### 2.1. Study Design

The analyses are based on the data from a cross-sectional survey carried out amongst consumers who shopped in grocery stores and shopping malls across Ankara, Turkey from April 2019 to April 2021. The study embraced a mixture of regression model and class-membership equation. Body mass index (BMI) was afterwards calculated by the researchers from adjusted body weight and height with unit kg/m^2^. The questionnaires referred to for measuring nutrition knowledge and nutrition literacy level were applied to the participants.

### 2.2. Participants

Subject participation was voluntary. To be eligible for the study, participants had to be 19–64 years old, and there were no other exclusion criteria. The Ethics Board of Scientific Research and Publication approved the study. All subjects provided written informed consent, and all procedures were under the ethical standards described in the Declaration of Helsinki. The interviews, which were resorted to as data collection media, ranged in duration from 25 to 60 min. The study was approved by the Scientific Research and Publication Ethics Committee of Ankara Medipol University of Turkey (Ethics Approval No 50).

### 2.3. Instruments

#### 2.3.1. General Nutrition Knowledge Questionnaire (GNKQ)

Nutrition knowledge level was assessed by the GNKQ developed by Parmenter and Wardle [16,17]. The GNKQ comprises 4 multiple-choice options in 4 main sections containing 127 items: Dietary guideline recommendations (11 items) (A1), sources of nutrients (70 items) (A2), everyday food choices (11 items) (A3) and diet–disease relationship (35 items) (A4). Participants answered one of the 4 different options: “more, same, less, not sure”, “yes, no, not sure”, “high, low, not sure”, “agree, disagree, not sure”. The overall internal consistency on the scale was found as high (Cronbach’s alpha = 0.70–0.97) [17].

#### 2.3.2. Evaluation Instrument of Nutrition Literacy on Adults (EINLA)

Nutrition literacy level was evaluated with the EINLA, which involves five subgroups containing 35 questions: General nutrition knowledge (10 questions) (B1), reading comprehension (6 questions) (B2), food groups (10 questions) (B3), portion quantities (3 questions) (B4) and numerical literacy and food label reading (6 questions) (B5). “One point” was given to the correct questions and “zero points” to the questions that were answered incorrectly or left blank. In the subgroups of B1 and B3, 0–3 points refer to insufficient, 4–7 points refer to borderline, and 8–10 points refer to sufficient; in the subgroups of B2 and B5, 0–2 points mean insufficient, 3–4 points mean borderline, and 5–6 points mean sufficient; in the B4 subgroup, 0–1 point indicates insufficient, 2 points indicate borderline, and 3 points indicate sufficient nutrition literacy. Out of the total score, 0–11 points are classified as insufficient, 12–23 points borderline and 24–35 points as sufficient nutrition literacy levels. The Cronbach’s alpha reliability coefficient of the assessment tool was 0.75 [18].

### 2.4. Statistical Analyses

Statistical analyses were performed with the Statistical Package for Social Sciences (SPSS) software version 22. Continuous variables were demonstrated as the mean with standard deviation (SD), and categorical variables were demonstrated as the frequency with percentages. Differences between independent groups were compared with an independent sample *t*-test, and differences between three different groups were compared with one-way ANOVA for continuous preferences. Variables were measured with Pearson correlation coefficients. Multivariate logistic regression analysis was conducted using the selection of factors linked (*p* ≤ 0.20) with nutritional literacy in univariate analysis. The goodness of fit of multivariate logistic regression models was studied using the Hosmer–Lemeshow test. Odds ratios (ORs) and 95% confidence intervals (CIs) were presented. A *p*-value of less than 0.05 was statistically important.

## 3. Results

In the study, 58.4% of the participants are female, and the average age of all participants is 28.2 ± 10.9 years. More than half of the participants are university graduates (57.4%) and single (66.7%). Moreover, 26.5% of the participants are individuals who are overweight, and 7.3% are obese. The participants were enquired about their skipping of main meals and snacking habits. As per their responses, 47.4% of the participants appeared to be skipping lunch, and 46.1% of the participants seemed to be skipping breakfast. It was also discovered that 36.6% snacked twice a day, and 64.1% consumed three main meals (Table 1).

When the nutrition knowledge scores of the participants were delved into, referring to the General Nutrition Knowledge Questionnaire (GNKQ), all subgroup scores and total scores were significantly higher in females compared to males (*p* < 0.001). Singles and students scored higher in all subgroups of the GNKQ (*p* < 0.05). As the education level increased, so did the scores that were obtained from all the subgroups of the GNKQ (*p* < 0.001). Participants who did not skip main meals scored higher than those who did (*p* < 0.05). Participants with a healthy BMI range (*p* < 0.05) and participants who received nutrition education scored higher in all the subgroups of the GNKQ (*p* < 0.001). Aside from these, those who received nutrition education at school scored higher compared to those who received such education via attending a relevant course/seminar (*p* < 0.05) (Table 2). The EINLA subgroups and total scores of the participants are shown in Table 2. Females scored higher than males in the EINLA subgroups and total scores (*p* < 0.05). As the education level increased, so did the nutrition literacy score (*p* < 0.001). Singles scored higher compared to married persons (*p* < 0.001). The participants who skipped the main meal received a lower total score on the EINLA than those who did not (*p* < 0.001). The participants with a healthy BMI range scored the highest, whereas the participants who were obese scored the lowest (*p* < 0.001). The ones who obtained nutrition education had higher scores in all the subgroups of the EINLA when compared to those who did not (*p* < 0.001). Lastly, participants who received nutrition education at school scored higher than those who attended a related course/seminar (*p* < 0.001) (Table 2).

The results of univariate and multivariable logistic regression are demonstrated in Table 3. Age, gender, marital status, education status, employment status, BMI and nutrition education were significantly associated with nutrition literacy. Nutritional literacy risk factors were analyzed using multivariate logistic regression analysis. Age (OR = 0.971, 95% CI: 0.953–0.990) and nutrition knowledge (OR = 1.076, 95% CI: 1.061–1.091) were important risk factors for nutrition literacy (*p* < 0.001).

An important negative correlation was detected between age and general nutrition knowledge (r = −0.096; *p* < 0.001), reading comprehension (r = −0.085; *p* < 0.001), portion quantities (r = −0.113, *p* < 0.001) and numerical literacy and food label reading (r = −0.219; *p* < 0.001) subgroups’ scores and total scores (r = −0.181; *p* < 0.001). As BMI increased, general nutrition knowledge (r = −0.119; *p* < 0.001), reading comprehension (r = −0.105; *p* < 0.001), portion quantities (r = −0.106, *p* < 0.001) and numerical literacy and food label reading (r = −0.176, *p* < 0.001) subgroups’ scores and total scores (r = −0.174, *p* < 0.001) decreased (Table 4).

**Figure 1:**
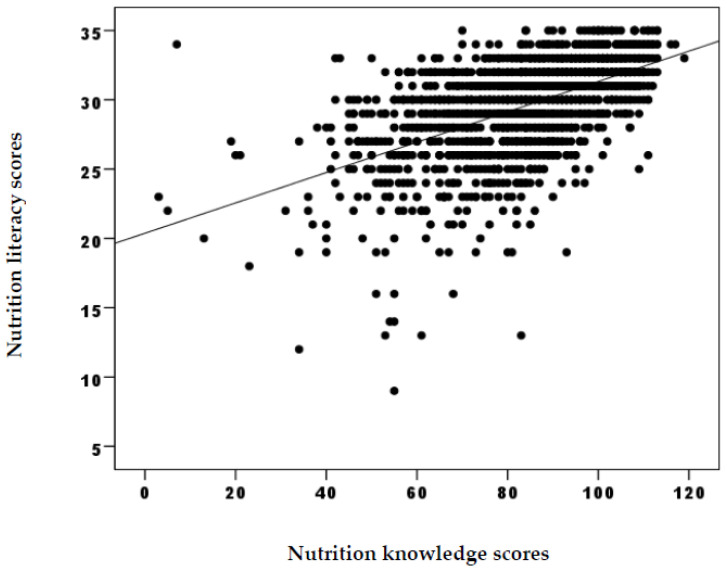
Correlation between nutrition knowledge and nutrition literacy scores.

Nutrition knowledge and general nutrition knowledge (r = 0.406; *p* < 0.001), reading comprehension (r = 0.290; *p* < 0.001), food groups (r = 0.162; *p* < 0.001), portion quantities (r = 0.265; *p*< 0.001) and numerical literacy and food label reading (r = 0.466; *p* < 0.001) subgroups’ scores bore an important positive correlation (Table 4). A significant positive correlation was spotted between the total scores of nutrition knowledge and nutrition literacy (r = 0.556; *p* < 0.001) (Figure 1).

## 4. Discussion

The total GNKQ score was higher amongst all the participants for the females, singles, those with a high education level, those with a healthy BMI range, those who did not skip the main meal and those who received nutrition education at school (*p* < 0.001). A study, which also made use of the GNKQ to evaluate nutrition knowledge, declared that young men had the lowest GNKQ scores [18]. In another study from Australia, the GNKQ score was positively associated with the level of education, which is also in line with the findings of the present study [19]. However, it is pinpointed that individuals’ nutritional knowledge alone does not alter their nutritional behavior, involving the behaviors that are pertinent to food choices [20]. To that end, there emerges a demand to shift beyond nutritional knowledge to broader constructs such as nutrition literacy to be able to gain the potential to further influence positive and desired changes in related behavior, such as the ones that entail food choices. The prevalence of individuals with sufficient nutrition literacy is indeed conflicting in the literature. Almost all of the respondents (94.6%) had sufficient nutrition literacy in the current study. In a study by Zoellner et al. (2009), 48.0% of the participants were announced to have adequate nutrition literacy, and in another study by Michou et al. (2019), 90.0% of the participants were announced to have adequate nutrition literacy [6,21]. In another study on 368 nurses in China, it was shared that 68.0% of the participants had borderline nutrition literacy [22]. In the present research, women had higher nutrition literacy scores than men (*p* < 0.001). This may be due to the fact that women cook more than men as part of their expected social responsibilities and attributed gender roles that, in turn, make these individuals mainly accountable for the nutrition of the household.

The line of the literature reveals more research on the issue. To cite an example, in the study by Aihara and Mina (2011) on the nutrition literacy levels of the elderly Japanese people, it was declared that only 30.7% of the participants had sufficient nutrition literacy [23]. The consumers’ low education level and advanced age were identified as negatively correlated with nutrition literacy scores, albeit the participants did not have trouble reading the energy and sugar content of the food on the food labels; it was underpinned that they had difficulty determining the portion size [22]. Middle-income individuals are witnessed to have higher nutritional literacy, and higher nutritional literacy is positively correlated with education level [23]. In addition, age, gender, marital status, education level and nutrition education were tracked down to be significantly linked to nutritional literacy. The participants with a university degree were found to have a 4.558 times higher nutrition literacy score (OR: 4.558, 95% CI: 2.469–8.413, *p* < 0.001). As can be understood from these outcomes, the education level of individuals is in a way mirrored in their nutrition literacy. It has also been suggested in the relevant line of literature that the dietary habits of individuals may be affected by their nutrition literacy. In fact, it is disclosed that the lower the health literacy scores in the low-income rural population, the lower the quality of the diet [21]. In a study on adults in the United States, the participants with insufficient nutrition literacy consumed more foods associated with the Western diet; conversely, those with higher nutrition literacy consumed more foods linked with the Mediterranean diet. It was, therefore, reported that nutrition literacy is effective in predicting dietary patterns as well [24].

It is pronounced that dietitians experience issues with conveying nutrition education to people with low FNL [25]. It was also confirmed that nutrition literacy can impact the ability to learn and apply the self-monitoring skills required to adopt and sustain a low-energy diet, which is a chief factor affecting sustainable weight loss. In a study conducted with individuals with obesity and persons who were overweight participating in a bodyweight loss program, the nutrition literacy scores of the participants with low education levels were revealed to be lower. With that being said, the participants with high nutrition literacy lost more weight within the six-month period, and their diet quality was higher [26]. In a study in New Zealand, it was alleged that nutrition literacy significantly affects anthropometric measurement and blood lipid level. Nutrition literacy score was inversely proportional to anthropometric measurements and total cholesterol/HDL-C ratio and positively correlated with high-density lipoprotein cholesterol (HDL-C) [27]. In this study, despite the fact that the diet quality or blood analysis was not among the factors analyzed, we proclaimed that the participants with a healthy BMI range had higher nutrition literacy (*p* < 0.001). Along with that, it is not yet clear if a healthy BMI range is the result or the cause of high nutrition literacy. Studies have hitherto manifested that insufficient health literacy is linked to poor disease knowledge, less self-management behavior, inadequate glycemic control and higher health care costs [28]. In addition, even though there is a significant positive relationship between health literacy and disease knowledge, it is discerned that insufficient knowledge level affects the self-management of disease [29]. Therefore, the gravity of nutrition literacy, which is actually a factor of health literacy, in the prevention and management of NCDs becomes even more evident. In another study carried out on elementary school students, it was unveiled that there is a positive correlation between nutrition and food literacy and diet variety. Children with low nutrition and food literacy had lower intakes of dietary protein, calcium, niacin, pyridoxine and folate [30].

It has also been divulged that the nutrition literacy of young adults impacts an individual’s facility to successfully grasp any salient information belonging to food labels, food choices, healthy cooking methods, dietary recommendations and food safety measures [31,32]. Accordingly, components such as nutrition knowledge and food preparation skills have positive effects on food intake in adults [33]. It was investigated that, in most cases, consumers were disordered when attempting to make healthy food choices due to possessing restricted or inaccurate nutrition knowledge [34]. The role of reading labels stands out at this very point since doing so enables the consumer to make more healthy food choices. There exist several and, as a matter of fact, conflicting findings in regard to the relationship between food label use and health literacy. In a study carried out in Canada, a positive relationship was established between food label use and health literacy [35]. In another study, an opposite relationship was condemned between health literacy and food label use [36]. Apart from these, in a different study, the participants uttered that they thought the food labels were merely for individuals with specific nutritional needs, and they were not engaged in these at the time of purchase and/or did not read the food labels “because they were starving” [37]. In this study, the food label reading score, which was one of the subgroups of nutrition literacy, was significantly higher in women, university graduates, those with a healthy BMI range and the participants who received nutrition education. Adults’ full understanding of the food label, which requires nutrition literacy, helps them select a healthy diet; however, a study shared no relationship between nutrition literacy and body mass index [38]. Nutrition literacy is considered essential to improve food label reading and full comprehension of the label for consumers. What is more, lately, scholars have been italicizing the growing demand for boosting both financial and digital literacy in individuals by offering novel models of mindsets for food and nutrition that can comply with the recent shifts in lifestyles and mentalities [39].

In the study, it was revealed that those who received nutrition education had higher nutrition knowledge and nutrition literacy than those who did not (*p* < 0.001). In spite of the fact that nutrition literacy is more of an umbrella term consisting of nutrition knowledge in this research, it was elucidated that there is an important positive correlation between nutrition knowledge and nutrition literacy (r = 0.556, *p* < 0.001). One can comfortably put forth that nutrition literacy can be achieved through obtaining the right forms of nutrition knowledge from the right sources. All the studies taken together [40] support the need for nutrition education at school and underscore the importance of gaining nutrition knowledge toward developing nutrition literacy. Undeniably, receiving nutrition information is a relatively easier job for adults, and the focal point needs to be on the application of nutritional information or dietary advice on a daily basis. In doing so, ensuring a healthy diet and developing a more critical stance towards available nutritional information and/or dietary advice obtained can become possible by taking into consideration unique nutritional needs at individual levels [41].

It would be fair to say that the exclusive features of this research are the size of the sample and the use of reliable and validated scales. Thinking that studies rarely encompass nutrition literacy criteria, this study’s focus on nutrition literacy is conceivably one of its pivotal strengths. On the flip side, this research has its own limitations. As the sample of this study covers consumers only from Ankara, the capital city of Turkey, the results cannot be generalized across the country or beyond. Particularly bearing in mind that Ankara, as the capital city, holds individuals that are relatively more intellectual and educated compared to rural areas, more studies can shed light on the situations with different profiles and unique cases, such as students with special needs, who all are engaged in programs covering healthy dieting and nutrition. This study is fulfilled as a cross-sectional study; therefore, there are inherent structural–methodological limitations as well in interpreting the cause-and-effect relationships.

## 5. Conclusions

The level of nutrition literacy affects how people seek and rely on nutrition knowledge. Understanding the causes and consequences of insufficient nutrition literacy is an important step towards reducing the burden of NCDs. It is known that insufficient nutrition literacy is a barrier to healthy eating. In this context, emphasis should be placed on increasing consumers’ awareness. To do such public health strategies and regulations that focus on increasing nutrition literacy, knowledge and health awareness in society should be evaluated for health practitioners and policymakers. Improving the dietary habits of society is a social and multifaceted task that requires understanding individuals’ food-related skills and abilities. While the use of food labels by nutritionally illiterate consumers and efforts to raise consumer awareness seem to be working, the issues related to label understanding can be more difficult to address. Some nutrition literacy interventions show promise in increasing the understanding and use of food labels. However, more studies are needed to determine the extent to which the findings can be generalized to other contexts. The effects of these interventions on both short and long-term nutritional behavior(s) and health outcomes should be examined using research designs that allow causal inferences or by controlling potential variables.

## Figures and Tables

**Table 1:** Demographic and anthropometric characteristics of the participants (*n* = 1600).

Variables	*n*	%
Gender		
Male	666	41.6
Female	934	58.4
Age (years) ($\bar{X}$± SD)	28.2 ± 10.9	
Marital status		
Single	1068	66.7
Married	532	33.3
Education status		
Primary school graduate	131	8.2
Middle/high school graduate	551	34.4
University graduate/postgraduate education	918	57.4
Employment status		
Employee	540	33.8
Unemployed	1060	66.2
Working status		
Student	864	54.0
Officer	229	14.3
Self-employed	187	11.7
Employee	164	10.3
Retired/housewife	156	9.7
Nutrition education		
Yes	361	22.6
No	1239	77.4
Nutrition education source		
School	314	87.0
Course/seminar	47	13.0
Body weight (kg) ($\bar{X}$± SD)	67.7 ± 13.8	
Height (cm) ($\bar{X}$± SD)	168.4 ± 8.5	
BMI (kg/m^2^)		
<18.5	90	5.6
18.5–24.9	969	60.6
25–29.9	424	26.5
≥30	117	7.3

**Table 2:** The General Nutrition Knowledge Questionnaire (GNKQ) and Evaluation Instrument of Nutrition Literacy on Adults (EINLA) scores ($\bar{X}$± SD).

	A1	A2	A3	A4	Total	B1	B2	B3	B4	B5	Total
Gender											
Male	6.3 ± 1.9	43.9 ± 10.4	7.1 ± 2.4	23.1 ± 5.8	80.5 ± 17.1	8.3 ± 1.5	5.2 ± 0.9	9.4 ± 1.3	1.6 ± 0.9	4.4 ± 1.4	29.0 ± 3.6
Female	6.8 ± 1.9	47.6 ± 9.9	7.8 ± 2.4	24.1 ± 5.9	86.3 ± 17.0	8.8 ± 1.3	5.3 ± 0.8	9.4 ± 0.9	1.8 ± 0.8	4.6 ± 1.5	30.0 ± 3.2
*p* value	<0.001	<0.001	<0.001	<0.001	<0.001	<0.001	0.001	0.119	0.001	0.033	<0.001
Marital status											
Single	6.7 ± 2.0	47.1 ± 10.3	7.6 ± 2.6	24.2 ± 5.9	85.6 ± 17.7	8.7 ± 1.4	5.3 ± 0.8	9.4 ± 1.1	1.8 ± 0.8	4.7 ± 1.4	29.9 ± 3.3
Married	6.4 ± 1.8	43.8 ± 9.8	7.3 ± 2.1	22.8 ± 5.8	80.3 ± 15.8	8.5 ± 1.4	5.2 ± 0.9	9.4 ± 1.2	1.6 ± 0.9	4.1 ± 1.5	28.8 ± 3.5
*p* value	0.026	<0.001	0.011	<0.001	<0.001	0.033	0.001	0.462	<0.001	<0.001	<0.001
Education level											
Primary school graduate	6.1 ± 1.7	40.5 ± 9.3	6.7 ± 2.0	20.5 ± 6.4	73.7 ± 15.3	7.9 ± 1.5	4.8 ± 1.0	9.3 ± 1.3	1.6 ± 0.9	3.4 ± 1.7	26.9 ± 3.7^a^
Middle/high school graduate	6.4 ± 1.9	45.1 ± 10.4	7.3 ± 2.4	23.1 ± 5.9	82.0 ± 17.3	8.4 ± 1.5	5.2 ± 0.9	9.4 ± 1.2	1.7 ± 0.9	4.5 ± 1.4	29.2 ± 3.5
University graduate/postgraduate	6.7 ± 2.0	47.4 ± 10.0	7.8 ± 2.4	24.6 ± 5.6	86.4 ± 16.9	8.9 ± 1.2	5.4 ± 0.8	9.4 ± 1.0	1.8 ± 0.8	4.7± 1.4	30.1 ± 3.1
*p* value	<0.001	<0.001	<0.001	<0.001	<0.001	<0.001	<0.001	0.682	0.015	<0.001	<0.001
Employment status											
Employee	6.4 ± 1.8	44.0 ± 10.4	7.3 ± 2.3	23.4 ± 5.9	81.1 ± 16.7	8.5 ± 1.4	5.2 ± 0.9	9.5 ± 1.1	1.6 ± 0.9	4.4 ± 1.5	29.1 ± 3.5
Unemployed	6.7 ± 2.0	47.1 ± 10.1	7.6 ± 2.5	23.9 ± 5.9	85.3 ± 17.4	8.7 ± 1.3	5.3 ± 0.8	9.4 ± 1.1	1.8 ± 0.8	4.6 ± 1.4	29.8 ± 3.3
*p* value	0.008	<0.001	0.004	0.075	<0.001	0.019	0.049	0.220	<0.001	0.001	<0.001
Working status											
Officer	6.4 ± 1.7	44.9 ± 9.5	7.4 ± 2.2	24.0 ± 5.1	82.8 ± 15.0	8.8 ± 1.3	5.4 ± 0.8	9.4 ± 1.1	1.6 ± 0.9	4.5 ± 1.4	29.7 ± 3.1
Worker	6.3 ± 1.8	43.5 ± 11.2	7.2 ± 2.4	22.0 ± 6.7	79.1 ± 18.3	8.2 ± 1.7	5.1 ± 1.0	9.5 ± 1.1	1.6 ± 0.9	4.1 ± 1.7	28.5 ± 3.7
Self-employed	6.6 ± 1.9	43.6 ± 10.4	7.4 ± 2.2	23.6 ± 5.6	81.2 ± 17.2	8.5 ± 1.5	5.0 ± 1.1	9.4 ± 1.2	1.6 ± 0.9	4.3 ± 1.5	28.9 ± 4.0
Retired/housewife	6.3 ± 1.8	43.6 ± 9.0	7.1 ± 2.0	22.1 ± 5.6	79.1 ± 14.7	8.3 ± 1.5	5.0 ± 1.0	9.4 ± 1.1	1.6 ± 0.9	3.6 ± 1.6	28.0 ± 3.6
Student	6.7 ± 2.0	47.8 ± 10.2	7.7 ± 2.6	24.3 ± 5.9	86.5 ± 17.6	8.8 ± 1.3	5.4 ± 0.8	9.4 ± 1.1	1.8 ± 0.8	4.8 ± 1.3	30.1 ± 3.1
*p* value	0.014	<0.001	0.011	<0.001	<0.001	<0.001	<0.001	0.012	<0.001	<0.001	<0.001
Skipping main meal											
Yes	6.4 ± 1.9	44.6 ± 10.4	7.2 ± 2.5	23.3 ± 5.8	81.5 ± 17.2	8.5 ± 1.5	5.2 ± 0.9	9.3 ± 1.2	1.7 ± 0.8	4.5 ± 1.4	29.1 ± 3.4
No	6.7 ± 2.0	46.9 ± 10.1	7.7 ± 2.4	24.0 ± 6.0	85.3 ± 17.2	8.7 ± 1.3	5.3 ± 0.9	9.4 ± 1.0	1.8 ± 0.9	4.6 ± 1.5	29.8 ± 3.4
*p* value	0.003	<0.001	<0.001	0.033	<0.001	<0.001	0.031	0.047	0.007	0.194	<0.001
BMI (kg/m^2^)											
<18.5	6.3 ± 2.2	46.7 ± 10.6	7.2 ± 2.7	23.3 ± 5.8	83.4 ± 18.8	8.8 ± 1.2	5.4 ± 0.8	9.2 ± 1.4	1.7 ± 0.9	4.6 ± 1.3	29.8 ± 3.1
18.5–24.9	6.7 ± 2.0	47.1 ± 10.4	7.7 ± 2.4	24.2 ± 5.9	85.7 ± 17.5	8.8 ± 1.3	5.3 ± 0.8	9.4 ± 1.1	1.8 ± 0.9	4.7 ± 1.4	30.0 ± 3.3
25–29.9	6.4 ± 1.8	44.3 ± 9.9	7.2 ± 2.3	23.0 ± 5.8	80.8 ± 16.4	8.4 ± 1.4	5.1 ± 1.0	9.4 ± 1.1	1.6 ± 0.9	4.3 ± 1.5	28.8 ± 3.4
≥30	6.4 ± 1.8	43.6 ± 9.5	7.1 ± 2.2	22.9 ± 6.2	80.0 ± 15.4	8.3 ± 1.4	5.2 ± 1.0	9.4 ± 1.2	1.5 ± 0.8	4.1 ± 1.6	28.4 ± 3.5
*p* value	0.005	<0.001	<0.001	0.002	<0.001	<0.001	<0.001	0.494	<0.001	<0.001	<0.001
Nutrition education											
Yes	7.8 ± 1.8	53.0 ± 8.6	9.0 ± 2.1	26.8 ± 4.8	96.5 ± 14.3	9.2 ± 1.2	5.5 ± 0.7	9.6 ± 0.7	2.0 ± 0.8	5.2 ± 1.2	31.5 ± 2.7
No	6.2 ± 1.9	44.0 ± 9.9	7.1 ± 2.3	22.8 ± 5.9	80.2 ± 16.3	8.5 ± 1.4	5.2 ± 0.9	9.3 ± 1.2	1.6 ± 0.9	4.3 ± 1.5	29.0 ± 3.4
*p* value	<0.001	<0.001	<0.001	<0.001	<0.001	<0.001	<0.001	<0.001	<0.001	<0.001	<0.001
Nutrition education resource											
School	7.8 ± 1.7	54.1 ± 7.9	9.1 ± 2.0	27.3 ± 4.2	98.4 ± 13.2	9.3 ± 1.1	5.5 ± 0.6	9.6 ± 0.7	2.0 ± 0.8	5.3 ± 1.0	31.7 ± 2.5
Course/seminar	7.3 ± 2.2	45.6 ± 9.2	8.3 ± 2.4	23.3 ± 6.8	84.4 ± 15.7	8.6 ± 1.6	5.1 ± 1.1	9.7 ± 0.6	2.1 ± 0.8	4.5 ± 1.5	30.0 ± 3.7
*p* value	0.091	<0.001	0.024	<0.001	<0.001	0.001	<0.001	0.310	0.455	<0.001	<0.001

GNKQ, A1–A4; A1: Dietary guideline recommendations, A2: Sources of nutrients, A3: Everyday food choices, A4: Diet–disease relationship. EINLA, B1–B5; B1: General nutrition knowledge, B2: Reading comprehension, B3: Food groups, B4: Portion quantities, B5: Numerical literacy and food label reading.

**Table 3:** Factors affecting nutrition literacy scores.

Variables	*n* (%)	Univariate Analysis			Multivariable Analysis		
		OR	95% CI	*p* Value	OR	95% CI	*p* Value
Age (years) ($\bar{X}$± SD)	28.2 ± 10.9	0.970	0.953–0.986	<0.001	0.971	0.953–0.990	0.002
Gender							
Male	666 (41.6)	1					
Female	934 (58.4)	1.658	1.072–2.564	0.023			
Marital status							
Single	1068 (66.7)	1					
Married	532 (33.3)	1.898	1.227–2.938	0.004			
Education							
Primary school graduate	131 (8.2)	1					
Middle/high school graduate	551 (34.4)	2.213	1.216–4.028	0.009			
University graduate/postgraduate	918 (57.4)	4.558	2.469–8.413	<0.001			
Employment status							
Employee	540 (33.8)	1					
Unemployed	1060 (66.2)	1.596	1.029–2.475	0.037			
Working status							
Official	229 (14.3)	1					
Worker	164 (10.3)	0.422	0.187–0.956	0.039			
Self-employed	187 (11.7)	0.457	0.204–1.023	0.057			
Retired/housewife	156 (9.7)	0.400	0.176–0.905	0.028			
Student	864 (54.0)	1.416	0.675–2.969	0.358			
Skipping main meal							
Yes	599 (37.4)	1					
No	1001 (62.6)	1.156	0.743–1.800	0.521			
BMI (kg/m^2^)							
<18.5	90 (5.6)	1					
18.5–24.9	969 (60.6)	1.468	0.708–3.044	0.303			
25–29.9	424 (26.5)	2.349	1.172–4.707	0.016			
≥30	117 (7.3)	1.453	0.516–4.090	0.479			
Nutrition education							
Yes	361 (22.6)	1					
No	1239 (77.4)	3.444	1.576–7.528	0.002			
Nutrition knowledge ($\bar{X}$± SD)	83.9 ± 17.3	1.075	1.060–1.089	<0.001	1.076	1.061–1.091	<0.001

**Table 4:** Correlation between nutritional literacy and age, BMI and nutrition knowledge of the participants.

		B1	B2	B3	B4	B5	Total
Age (years)	r	−0.096 **	−0.085 **	0.003	−0.113 **	−0.219 **	−0.181 **
	*p*	<0.001	0.001	0.895	<0.001	<0.001	<0.001
BMI (kg/m^2^)	r	−0.119 **	−0.105 **	0.008	−0.106 **	−0.176 **	−0.174 **
	*p*	<0.001	<0.001	0.752	<0.001	<0.001	<0.001
Nutrition knowledge	r	0.406 **	0.290 **	0.162 **	0.265 **	0.466 **	0.556 **
	*p*	<0.001	<0.001	<0.001	<0.001	<0.001	<0.001

B1: General nutrition knowledge, B2: Reading comprehension, B3: Food groups, B4: Portion quantities, B5: Numerical literacy and food label reading. ** *p* < 0.001.

## Data Availability

Data are available from the corresponding author.

## References

[1] Velardo S. (2015). The nuances of health literacy, nutrition literacy, and food literacy. J. Nutr. Educ. Behav..

[2] Sanlier N. (2009). The knowledge and practice of food safety by young and adult consumers. Food Control.

[3] Carrara A., Schulz P.J. (2017). The role of health literacy in predicting adherence to nutritional recommendations: A systematic review. Patient Educ. Couns..

[4] Johri M., Subramanian S.V., Koné G.K., Dudeja S., Chandra D., Minoyan N., Sylvestre M.-P., Pahwa S. (2016). Maternal health literacy is associated with early childhood nutritional status in India. J. Nutr..

[5] Madalı B., Dikmen D., Piyal B. (2017). Beslenme Bilgi Düzeyinin Değerlendirilmesinde Sağlık Okuryazarlığı Yeterli mi?. Beslenme Ve Diyet Derg..

[6] Zoellner J., Connell C., Bounds W., Crook L., Yadrick K. (2009). Nutrition literacy status and preferred nutrition communication channels among adults in the Lower Mississippi Delta. Prev. Chronic Dis..

[7] Carbone E.T., Zoellner J.M. (2012). Nutrition and health literacy: A systematic review to inform nutrition research and practice. J. Acad. Nutr. Diet..

[8] Gibbs H.D., Ellerbeck E.F., Gajewski B., Zhang C., Sullivan D.K. (2018). The Nutrition Literacy Assessment Instrument is a valid and reliable measure of nutrition literacy in adults with chronic disease. J. Nutr. Educ. Behav..

[9] Rothman R.L., Housam R., Weiss H., Davis D., Gregory R., Gebretsadik T., Shintani A., Elasy T.A. (2006). Patient understanding of food labels: The role of literacy and numeracy. Am. J. Prev. Med..

[10] Viswanathan M., Hastak M., Gau R. (2009). Understanding and facilitating the usage of nutritional labels by low-literate consumers. J. Public Policy Mark..

[11] Velardo S. (2017). Nutrition literacy for the health literate. J. Nutr. Educ. Behav..

[12] Silk K.J., Sherry J., Winn B., Keesecker N., Horodynski M.A., Sayir A. (2008). Increasing nutrition literacy: Testing the effectiveness of print, web site, and game modalities. J. Nutr. Educ. Behav..

[13] Azevedo Perry E., Thomas H., Samra H.R., Edmonstone S., Davidson L., Faulkner A., Petermann L., Manafò E., I Kirkpatrick S. (2017). Identifying attributes of food literacy: A scoping review. Public Health Nutr..

[14] Gibbs H., Chapman-Novakofski K. (2012). Exploring nutrition literacy: Attention to assessment and the skills clients need. Health.

[15] Aktas N., Ozdogan Y. (2016). A study of the state of knowing the nutritional literacy concept in Turkey. Res. World.

[16] Parmenter K., Wardle J. (1999). Development of a general nutrition knowledge questionnaire for adults. Eur. J. Clin. Nutr..

[17] Cesur B., Koçoğlu G., Sümer H. (2015). Evaluation instrument of nutrition literacy on adults (EINLA) A validity and reliability study. IFNM.

[18] Kullen C.J., Iredale L., Prvan T., O’Connor H.T. (2016). Evaluation of general nutrition knowledge in Australian military per-sonnel. J. Acad. Nutr. Diet..

[19] Iyer P., Beck E.J., Walton K.L. (2020). Exploring nutrition knowledge and dietary intake of adults with spinal cord injury in specialist rehabilitation. Spinal Cord.

[20] Vaitkeviciute R., Ball L.E., Harris N. (2015). The relationship between food literacy and dietary intake in adolescents: A system-atic review. Public Health Nutr..

[21] Michou M., Panagiotakos D.B., Lionis C., Costarelli V. (2019). Socioeconomic inequalities in relation to health and nutrition literacy in Greece. Int. J. Food Sci. Nutr..

[22] Law Q.P.S., Yau A.H.Y., Chung J.W.Y. (2019). Chinese adults’ nutrition label literacy in Hong Kong: Implications for nurses. Nurs. Health Sci..

[23] Aihara Y., Mina J. (2011). Barriers and catalysts of nutrition literacy among elderly Japanese people. Health Promot. Int..

[24] Taylor M.K., Sullivan D.K., Ellerbeck E.F., Gajewski B.J., Gibbs H.D. (2019). Nutrition literacy predicts adherence to healthy/unhealthy diet patterns in adults with a nutrition-related chronic condition. Public Health Nutr..

[25] Wood J., Gillis D.E. (2015). Exploring dietitians’ engagement with health literacy: Concept and practice. Can. J. Diet. Pract. Res..

[26] Rosenbaum D.L., Clark M.H., Convertino A.D., Call C.C., Forman E.M., Butryn M.L. (2018). Examination of nutrition lit-eracy and quality of self-monitoring in behavioral weight loss. Ann. Behav. Med..

[27] Mearns G.J., Chepulis L., Britnell S., Skinner K. (2017). Health and nutritional literacy of New Zealand nursing students. J. Nurs. Educ..

[28] Van der Heide I., Uiters E., Rademakers J., Struijs J.N., Schuit A.J., Baan C.A. (2017). Associations among health literacy, diabetes knowledge and self-management behavior in adults with diabetes: Results of a dutch cross-sectional study. J. Health Commun..

[29] Tseng H.M., Liao S.F., Wen Y.P., Chuang Y.J. (2017). Stages of change concept of the transtheoretical model for healthy eating links health literacy and diabetes knowledge to glycemic control in people with type 2 diabetes. Prim. Care Diabetes.

[30] Doustmohammadian A., Omidvar N., Keshavarz-Mohammadi N., Eini-Zinab H., Amini M., Abdollahi M., Haidari H. (2020). Low food and nutrition literacy (FNLIT): A barrier to dietary diversity and nutrient adequacy in school age children. BMC Res. Notes.

[31] Al Tell M., Natour N., Alshawish E., Badrasawi M. (2023). The relationship between nutrition literacy and nutrition information seeking attitudes and healthy eating patterns among a group of palestinians. BMC Public Health.

[32] Chung L.M.Y. (2017). Food literacy of adolescents as a predictor of their healthy eating and dietary quality. J. Child Adolesc. Behav..

[33] Laska M.N., Larson N.I., Neumark-Sztainer D., Story M. (2012). Does involvement in food preparation track from adolescence to young adulthood and is it associated with better dietary quality? Findings from a 10-year longitudinal study. Public Health Nutr..

[34] Spiteri Cornish L., Moraes C. (2015). The impact of consumer confusion on nutrition literacy and subsequent dietary behavior: The impact of consumer confusion on nutrition literacy. Psychol. Mark..

[35] Sinclair S., Hammond D., Goodman S. (2013). Sociodemographic differences in the comprehension of nutritional labels on food products. J. Nutr. Educ. Behav..

[36] Vijaykumar S., Lwin M.O., Chao J., Au C. (2013). Determinants of food label use among supermarket shoppers: A Singaporean perspective. J. Nutr. Educ. Behav..

[37] Yarmohammadi P., Morowatisharifabad M.A., Rahaei Z., Khayyatzadeh S.S., Madadizadeh F. (2020). Nutrition literacy and its related demographic factors among workers of Taraz Steel company, Chaharmahal and Bakhtiari, Iran. Front. Public Health.

[38] Koca B., Arkan G. (2020). The relationship between adolescents’ nutrition literacy and food habits and affecting factors. Public Health Nutr..

[39] Scazzocchio B., Varì R., d’Amore A., Chiarotti F., Del Papa S., Silenzi A., Gimigliano A., Giovannini C., Masella R. (2021). Promoting Health and Food Literacy through Nutrition Education at Schools: The Italian Experience with MaestraNatura Program. Nutrients.

[40] Stanley K.C., Harrigan P.B., Serrano E.L., Kraak V.I. (2022). A systematic scoping review of the literacy literature to develop a digital food and nutrition literacy model for low income adults to make healthy choices in the online food retail ecosys-tem to reduce obesity risk. Obes. Rev..

[41] Zhang Y., Sun Q., Zhang M., Liu H. (2022). Nutrition literacy measurement tool with multiple features for Chinese adults. Food Nutr. Bull..

